# Evaluation of the inhibitory effect of ivermectin on the growth of *Babesia* and *Theileria* parasites in vitro and in vivo

**DOI:** 10.1186/s41182-019-0171-8

**Published:** 2019-07-11

**Authors:** Gaber El-Saber Batiha, Amani Magdy Beshbishy, Dickson Stuart Tayebwa, Oluyomi Stephen Adeyemi, Naoaki Yokoyama, Ikuo Igarashi

**Affiliations:** 1 National Research Center for Protozoan Diseases, Obihiro University of Agriculture and Veterinary Medicine, Nishi 2-13, Inada-cho, Obihiro, Hokkaido 080-8555 Japan; 2 Department of Pharmacology and Therapeutics, Faculty of Veterinary Medicine, Damanhour University, Damanhour, El Beheira 22511 Egypt; 3 Research Center for Tick and Tick-Borne Diseases, College of Veterinary Medicine, Animal Resources and Biosecurity, Makerere University, PO Box 7062, Kampala, Uganda; 4 Medicinal Biochemistry, Nanomedicine and Toxicology Laboratory, Department of Biological Sciences, Landmark University, Omu-Aran, Kwara 251101 Nigeria

**Keywords:** Ivermectin, *Babesia*, *Theileria*, In vitro, In vivo

## Abstract

**Background:**

Treatment is the principle way to control and eliminate piroplasmosis. The search for new chemotherapy against *Babesia* and *Theileria* has become increasingly urgent due to parasite resistance to current drugs. Ivermectin (IVM) was the world’s first endectocide, capable of killing a wide variety of parasites and vectors, both inside and outside the body. It is currently authorized to treat onchocerciasis, lymphatic filariasis, strongyloidiasis, and scabies. The current study documented the efficacy of IVM on the growth of *Babesia* and *Theileria* in vitro and in vivo.

**Methods:**

The fluorescence-based assay was used for evaluating the inhibitory effect of IVM on four *Babesia* species, including *B*. *bovis*, *B*. *bigemina*, *B*. *divergens*, *B*. *caballi*, and *Theileria equi*, the combination with diminazene aceturate (DA), clofazimine (CF), and atovaquone (AQ) on in vitro cultures, and on the multiplication of a *B*. *microti*-infected mouse model. The cytotoxicity of compounds was tested on Madin–Darby bovine kidney (MDBK), mouse embryonic fibroblast (NIH/3 T3), and human foreskin fibroblast (HFF) cell lines.

**Results:**

The half-maximal inhibitory concentration (IC_50_) values determined for IVM against *B*. *bovis*, *B*. *bigemina*, *B*. *divergens*, *B*. *caballi*, and *T*. *equi* were 53.3 ± 4.8, 98.6 ± 5.7, 30.1 ± 2.2, 43.7 ± 3.7, and 90.1 ± 8.1 μM, respectively. Toxicity assays on MDBK, NIH/3 T3, and HFF cell lines showed that IVM affected the viability of cells with a half-maximal effective concentration (EC_50_) of 138.9 ± 4.9, 283.8 ± 3.6, and 287.5 ± 7.6 μM, respectively. In the in vivo experiment, IVM, when administered intraperitoneally at 4 mg/kg, significantly (*p* < 0.05) inhibited the growth of *B*. *microti* in mice by 63%. Furthermore, combination therapies of IVM–DA, IVM–AQ, and IVM–CF at a half dose reduced the peak parasitemia of *B*. *microti* by 83.7%, 76.5%, and 74.4%, respectively. Moreover, this study confirmed the absence of *B*. *microti* DNA in groups treated with combination chemotherapy of IVM + DA and IVM + AQ 49 days after infection.

**Conclusions:**

These findings suggest that IVM has the potential to be an alternative remedy for treating piroplasmosis.

**Electronic supplementary material:**

The online version of this article (10.1186/s41182-019-0171-8) contains supplementary material, which is available to authorized users.

## Background

Babesiosis is a malaria-like parasitic disease caused by *Babesia*, a genus of Apicomplexa [1]. *Babesia bovis*, *B*. *bigemina*, and *B*. *divergens* infect cattle, causing bovine babesiosis. In Europe, bovine babesiosis, known as red water fever, is mainly caused by *B*. *divergens* and is considered the most important tick-transmitted disease in cattle [2], while *B*. *caballi* and *Theileria equi* infect horses causing equine piroplasmosis [3]. Human babesiosis is uncommon; however, it is important as an emerging disease in the Northeastern and Midwestern United States and parts of Europe, and sporadic throughout the rest of the world [4]. The spectrum of human babesiosis is broad, ranging from an apparently silent infection to a fulminant, malaria-like disease resulting occasionally in death [5].

Treatment of babesiosis and theileriosis in animals depends on two common drugs, namely diminazene aceturate (DA) and imidocarb dipropionate, while human babesiosis has been managed with a combination of atovaquone (AQ) and azithromycin and a combination of clindamycin and quinine [6]. Recently, González et al. showed the failure, ineffectiveness, adverse reaction, and relapsing babesiosis of clindamycin and quinine combination treatment in splenectomized patients infected by *B*. *divergens* or *B*. *microti* [7], while Hatcher et al. showed that patients affected by severe babesiosis had adverse reactions to quinine treatment and persistently high parasitemia more than 10 days after treatment with a combination of AQ and azithromycin [8]. In addition, *Babesia gibsoni* has been shown to be resistant to AQ [9]. Therefore, research to find new drugs and drug targets is the fundamental approach for addressing current limitations.

Ivermectin (IVM) is a macrocyclic lactone derived from avermectin, which is produced by an *actinomycete*, *Streptomyces avermitilis* [10]. IVM is a safe drug active against a wide range of internal and external parasites, and it is used widely in both veterinary and human medicine [11, 12]. In human medicine, IVM is used to treat onchocerciasis (river blindness). IVM is effective against many worm infestations (such as strongyloidiasis, ascariasis, trichuriasis, filariasis, and enterobiasis) and some epidermal parasitic skin diseases, including scabies [13]. Originally thought to have antibacterial or antiviral properties, IVM has recently been reported to kill *Mycobacterium tuberculosis*, including multidrug-resistant strains [14]. IVM induces chloride-dependent membrane hyperpolarization and cell death in leukemia cells, prompting suggestions that it could be rapidly put into clinical trials for leukemia. IVM was shown to be a highly potent inhibitor of yellow fever virus replication and the replication of several other flaviviruses, notably dengue, Japanese encephalitis, and tick-borne encephalitis [14]. Recently, the endectocide IVM has arisen as a promising new tool to be added to malaria control programs [12]. Moreover, new possible uses are continuing to emerge, heralding potential breakthroughs in tackling various neglected tropical diseases—and beyond. Research has shown that, for human African trypanosomiasis (sleeping sickness), deworming cattle with single doses of IVM decrease the survival and fecundity of disease-transmitting tsetse flies feeding on cattle by up to 94% [15]. IVM is also efficacious in curing cutaneous leishmaniasis, killing *Leishmania* parasites in vitro and via subcutaneous inoculation [16].

In veterinary medicine, IVM is used against many intestinal worms, most mites, and some lice. It is sometimes administered in combination with other medications to treat a broad spectrum of animal parasites [10]. In addition, IVM can be given by mouth, topically, or via injection. As a drug targeting nematode and arthropod parasites, IVM has not been reported to directly interact with any mammalian proteins with high selectivity [17]. In this study, we evaluated the effects of IVM against the growth of *B*. *bigemina*, *B*. *bovis*, *B*. *divergens*, *B*. *caballi*, and *T*. *equi* in vitro as well as the chemotherapeutic potential of IVM on *B*. *microti* in vivo.

## Results

### The growth inhibitory effect of IVM against *Babesia* and *Theileria*

**Fig. 1:**
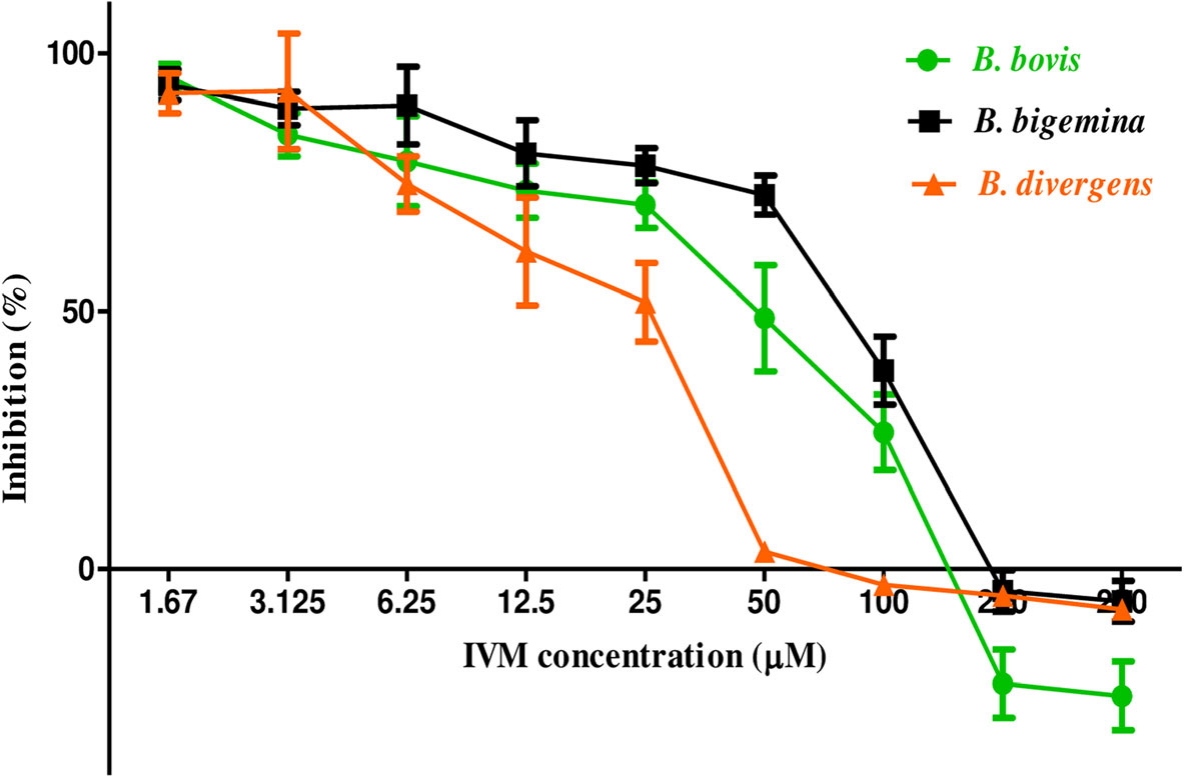
The dose-response curves of ivermectin against bovine *Babesia* parasites in vitro. The curve shows the correlation between relative fluorescence units (RFUs) and the log concentrations of IVM (μM) in *B*. *bovis*, *B*. *bigemina*, and *B*. *divergens* treated with various concentrations of IVM. The result was determined by fluorescence assay after 96 h of incubation. The values obtained from three separate trials were used to determine the IC_50_s using nonlinear regression (curve fitting analysis) in GraphPadPrism software

**Fig. 2:**
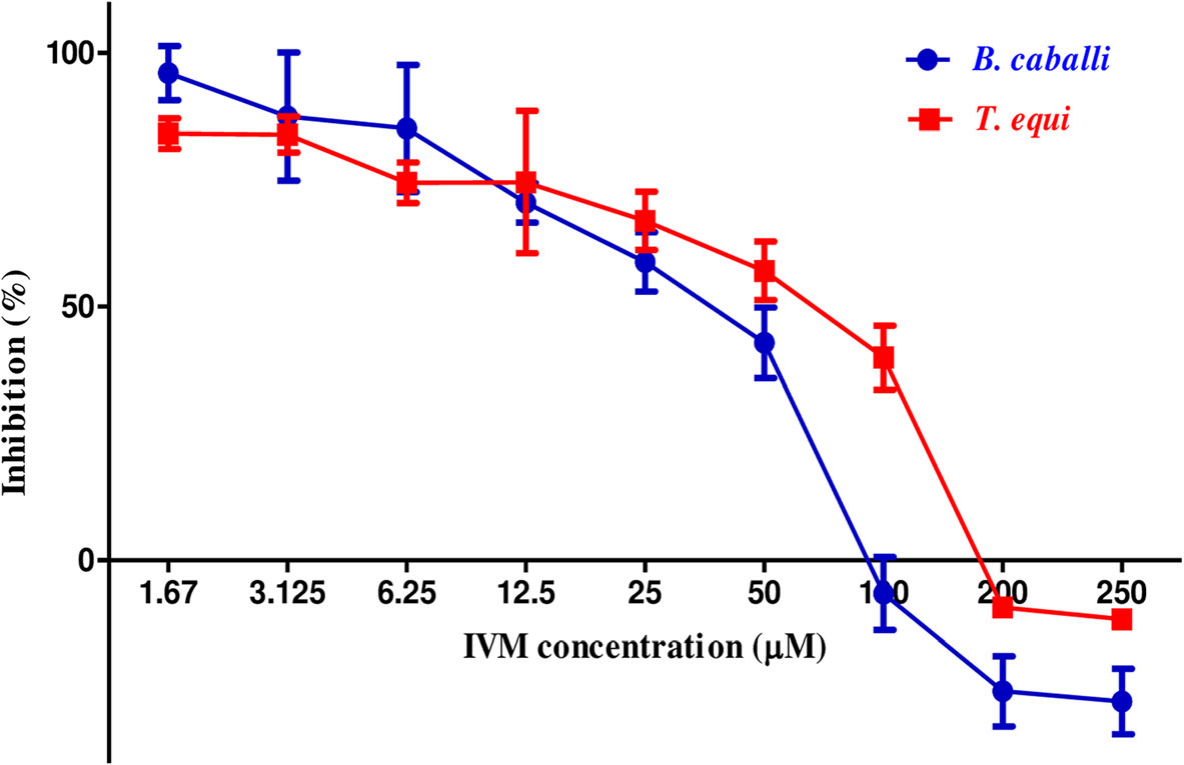
The dose-response curves of ivermectin against equine piroplasm parasites in vitro. The curve shows the correlation between relative fluorescence units (RFUs) and the log concentrations of IVM (μM) in *B*. *caballi* and *T*. *equi* treated with various concentrations of IVM. The result was determined by fluorescence assay after 96 h of incubation. The values obtained from three separate trials were used to determine the IC_50_s using nonlinear regression (curve fitting analysis) in GraphPadPrism software

Growth inhibitory assays were conducted on five species: *B*. *bovis*, *B*. *bigemina*, *B*. *divergens*, *B*. *caballi*, and *T*. *equi*. IVM inhibited the multiplication and growth of all species tested in a dose-dependent manner (Figs. 1 and 2). The half-maximal inhibitory concentration (IC_50_) values of IVM on *B*. *bovis*, *B*. *bigemina*, *B*. *divergens*, *B*. *caballi*, and *T*. *equi* were 53.3 ± 4.8, 98.6 ± 5.7, 30.1 ± 2.2, 43.7 ± 3.7, and 90.1 ± 8.1 μM, respectively (Table 1). In this study, diminazene aceturate (DA) showed IC_50_ values at 0.35, 0.68, 0.43, 0.022, and 0.71 μM against *B*. *bovis*, *B*. *bigemina*, *B*. *divergens*, *B*. *caballi*, and *T*. *equi*, respectively. Atovaquone (AQ) showed IC_50_ values at 0.039, 0.701, 0.038, 0.102, and 0.095 μM against *B*. *bovis*, *B*. *bigemina*, *B*. *divergens*, *B*. *caballi*, and *T*. *equi*, respectively. Clofazimine (CF) showed IC_50_ values at 8.24, 5.73, 13.85, 7.95, and 2.88 μM against *B*. *bovis*, *B*. *bigemina*, *B*. *divergens*, *B*. *caballi*, and *T*. *equi*, respectively **(**Additional file 3: Table S3). The effectiveness of IVM was not influenced by the diluent since there was no significant difference in the inhibition between wells containing DMSO and untreated wells. The precultivation of RBCs with IVM was conducted to determine its direct effect on host RBCs. Bovine and equine RBCs were incubated with IVM at 10, 100, and 200 μM for 3 and 6 h to be used for the parasite subculture. The multiplication of all parasites did not significantly differ between IVM-treated RBCs and normal RBCs for either species (data not shown).

**Table 1:** IC_50_ and selectivity index of IVM

Compound	*Babesia* and *Theileria*	IC_50_ (μM)^a^	EC_50_ (μM)^b^			Selective indices^c^		
			MDBK	NIH/3 T3	HFF	MDBK	NIH/3 T3	HFF
IVM	*B*. *bovis*	53.3 ± 4.8	138.9 ± 4.9	283.8 ± 3.6	287.5 ± 7.6	2.6	5.3	5.4
	*B*. *bigemina*	98.6 ± 5.7	138.9 ± 4.9	283.8 ± 3.6	287.5 ± 7.6	1.4	2.9	2.9
	*B*. *divergens*	30.1 ± 2.2	138.9 ± 4.9	283.8 ± 3.6	287.5 ± 7.6	4.6	9.4	9.6
	*B*. *caballi*	43.7 ± 3.7	138.9 ± 4.9	283.8 ± 3.6	287.5 ± 7.6	3.2	6.5	6.6
	*T*. *equi*	90.1 ± 8.1	138.9 ± 4.9	283.8 ± 3.6	287.5 ± 7.6	1.5	3.1	3.2

^a^Half-maximal inhibition concentration of ivermectin on the in vitro culture of parasites. The value was determined from the dose-response curve using nonlinear regression (curve fit analysis). The values are the means of triplicate experiments

^b^Half-maximal effective concentration of ivermectin on cell lines. The values were determined from the dose-response curve using nonlinear regression (curve fit analysis). The values are the means of triplicate experiments

^c^Ratio of the EC_50_ of cell lines to the IC_50_ of each species. High numbers are favorable

*IVM* ivermectin, *MDBK* Madin–Darby bovine kidney, *NIH*/*3 T3* Mouse embryonic fibroblast, *HFF* Human foreskin fibroblast

### Toxicity of IVM, DA, AQ, and CF on MDBK, NIH/3 T3, and HFF cell lines

IVM showed an inhibitory effect on the in vitro culture of *Babesia* and *Theileria* parasites. Therefore, the effect of IVM on host cells was evaluated using Madin–Darby bovine kidney (MDBK), NIH/3 T3, and human foreskin fibroblast (HFF) cell lines to see the cytotoxicity of the IVM compound (Table 1). The half-maximal effective concentration (EC_50_) values of IVM on MDBK, NIH/3 T3, and HFF cell lines were 138.9 ± 4.9, 283.8 ± 3.6, and 287.5 ± 7.6 μM, respectively. The selectivity indexes are defined as the ratio of cell line EC_50_ to the parasite IC_50_. The highest selectivity index was achieved on *B*. *divergens*; as in the case of the MDBK cell line, it was found to be 4.6 times higher than the IC_50_; while in case of the NIH/3 T3 cell line, it was found to be 9.4 times higher than the IC_50_; and in case of the HFF cell line, it was found to be 9.6 times higher than the IC_50_ (Table 1). In a separate assay, DA and AQ at concentrations of 100 μM did not show any inhibition of MDBK, NIH/3 T3, and HFF cell viability, while CF showed inhibition only on MDBK with an EC_50_ value at 34 ± 3.4 μM (Additional file 3: Table S3). The highest selectivity index of DA was achieved on *B*. *caballi*, while for AQ and CF the highest selectivity index was achieved against *B*. *divergens* and *T*. *equi*, respectively (Additional file 3: Table S3).

### The viability of parasites treated with IVM

A viability assay was performed to determine whether the concentrations of IVM could completely clear parasites after 4 days of successive treatment, followed by withdrawal of the drug pressure. *B*. *bovis*, *B*. *bigemina*, *B*. *divergens*, and *B*. *caballi* treated with IVM could not regrow at the concentration of 4 × IC_50_, while *T*. *equi* could regrow at the concentration of 4 × IC_50_ (Table 2).

**Table 2:** The viability of *Babesia* and *Theileria* parasites treated with IVM

Drugs	Conc. of compounds	Parasites				
		*B*. *bovis*	*B*. *bigemina*	*B*. *divergens*	*B*. *caballi*	*T*. *equi*
IVM	0.25 × IC_50_	**+**	**+**	**+**	**+**	**+**
	0.5 × IC_50_	**+**	**+**	**+**	**+**	**+**
	1 × IC_50_	**+**	**+**	**+**	**+**	**+**
	2 × IC_50_	**+**	**+**	**+**	**+**	**+**
	4 × IC_50_	**–**	**–**	**–**	**–**	**+**
	Untreated control	**+**	**+**	**+**	**+**	**+**

The positive (+) shows the regrowth of parasites, and the negative (−) shows the total clearance of parasites on day 8 after withdrawing the drug pressure

### The effects of the combination of IVM with DA, AQ, and CF in vitro

The drug combination assay was performed to examine whether the combined treatments are synergism (give a greater effect), additive (similar effect), or antagonism (reduce or block the effect). Five selected concentrations of IVM, as recommended in the Chou–Talalay method [18], were combined with DA, AQ, and CF at a constant ratio of (1:1). The percentage of inhibition of the single drug and each combination was analyzed using CompuSyn software to generate the combination index (CI) value at IC_50_, IC_75_, IC_90_, and IC_95_ (Additional file 2: Table S2). The drug combination effect was considered synergetic if the value was less than 0.90, additive if the value was at a range of 0.90–1.10, and antagonistic if the value was more than 1.10. The effects of combination treatment of IVM-DA, IVM-AQ, and IVM-CF against *B*. *bovis*, *B*. *bigemina*, *B*. *divergens*, *B*. *caballi*, and *T*. *equi* are shown in Table 3. The combination treatments of IVM-DA showed synergistic effects against *B*. *bigemina*, *B*. *divergens*, and *B*. *caballi*, and an additive effect against *B*. *bovis* and *T*. *equi*. The combination treatments of IVM-AQ showed a synergistic effect against *B*. *bigemina* but an additive effect against *B*. *bovis*, *B*. *divergens*, *B*. *caballi*, and *T*. *equi*. Combination treatments of IVM-CF showed additive effects against *B*. *bovis*, *B*. *bigemina*, *B*. *divergens*, *B*. *caballi*, and *T*. *equi*, while none of the combinations showed an antagonistic effect.

**Table 3:** The effect of ivermectin with diminazene aceturate, atovaquone, and clofazimine against *Babesia* and *Theileria* parasites in vitro

Drug combinations		Parasites				
		*B*. *bovis*	*B*. *bigemina*	*B*. *divergens*	*B*. *caballi*	*T*. *equi*
IVM + DA	CI values	1.11051	0.40539	0.01779	0.14328	1.10124
	Degree of association	Additive	Synergistic	Synergistic	Synergistic	Additive
IVM + AQ	CI values	1.04737	0.82283	1.04901	1.07398	1.05367
	Degree of association	Additive	Synergistic	Additive	Additive	Additive
IVM + CF	CI values	0.92317	1.01390	1.01372	1.10847	0.99644
	Degree of association	Additive	Additive	Additive	Additive	Additive

*CI* combination index, *AQ* atovaquone, CF clofazimine, *DA* diminazene aceturate

### The chemotherapeutic effect of IVM against *B*. *microti*

**Fig. 3:**
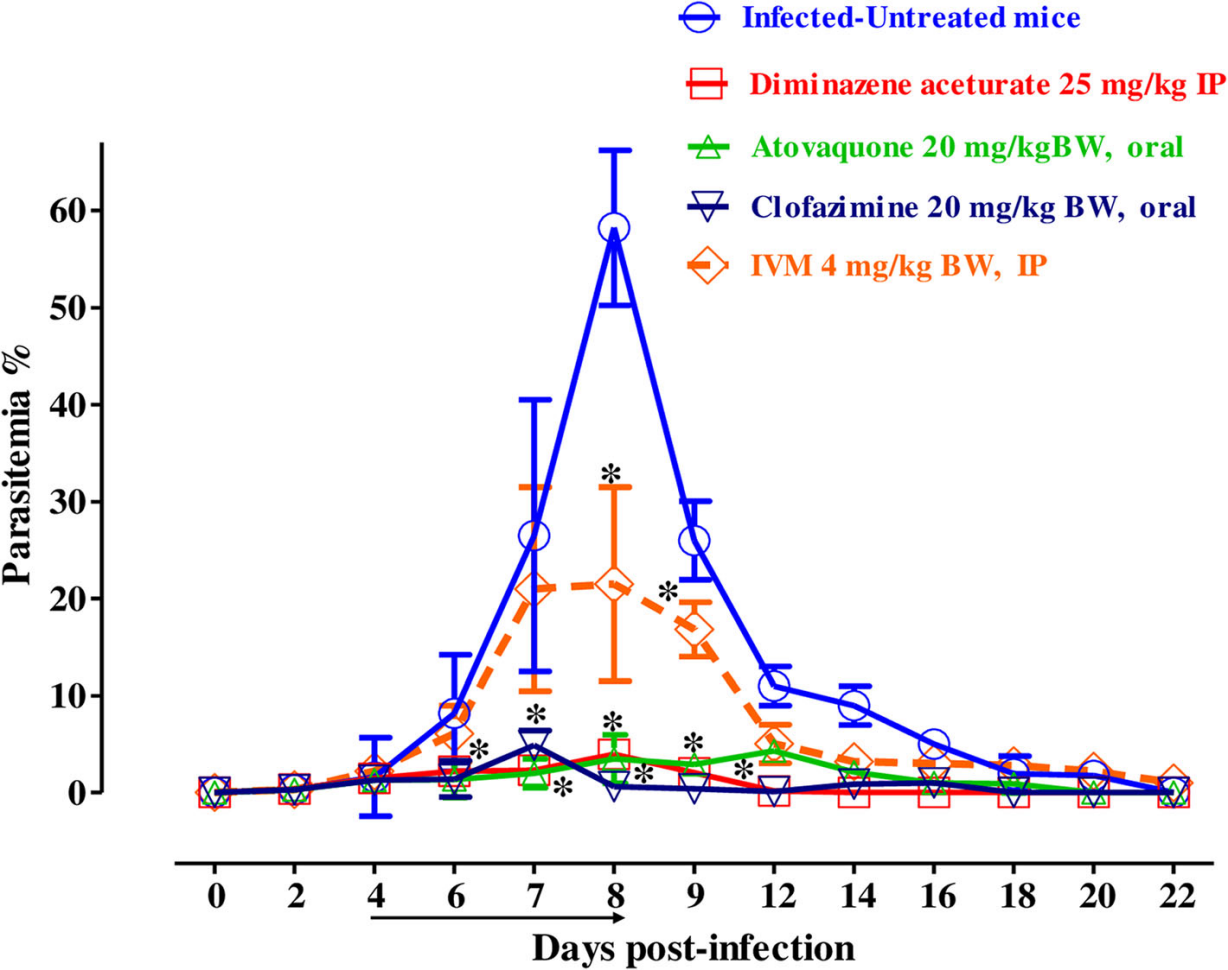
The growth inhibition of IVM on *B*. *microti* in vivo**.** Graph showing the inhibitory effects of DA-IP, AQ-oral, CF-oral, IVM-IP treatment as compared to the untreated group. The values plotted indicate the mean ± standard deviation for two separate experiments. Asterisks (*) indicate statistical significance (*p* < 0.05) based on one-way ANOVA Tukey’s test using GraphPad Prism version 5.0 for Windows (GraphPad Software Inc., San Diego, CA, USA). The arrow indicates five consecutive days of treatment. Parasitemia was calculated by counting infected RBCs among 2000 RBCs using Giemsa-stained thin blood smears

**Fig. 4:**
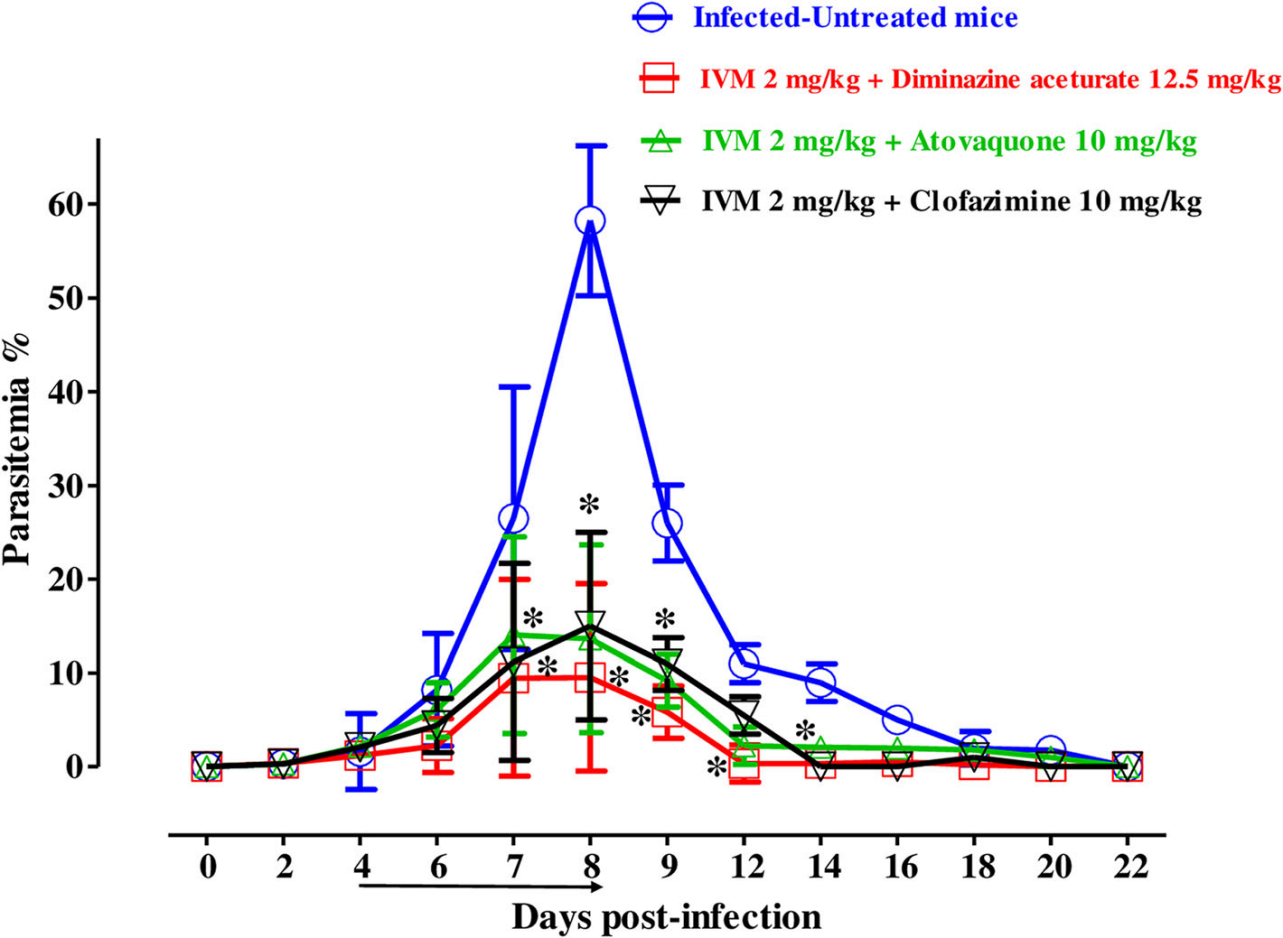
The growth inhibition of IVM combinations on *B*. *microti* in vivo. The graph shows the inhibitory effects of DA, AQ, and CF combined with IVM treatments as compared with the untreated group. The values plotted indicate the mean ± standard deviation for two separate experiments. The asterisks (*) indicate statistical significance (*p* < 0.05) based on one-way ANOVA Tukey’s test using GraphPad Prism version 5.0 for Windows (GraphPad Software Inc., San Diego, CA, USA). The arrow indicates five consecutive days of treatment. Parasitemia was calculated by counting infected RBCs among 2000 RBCs using Giemsa-stained thin blood smears

**Fig. 5:**
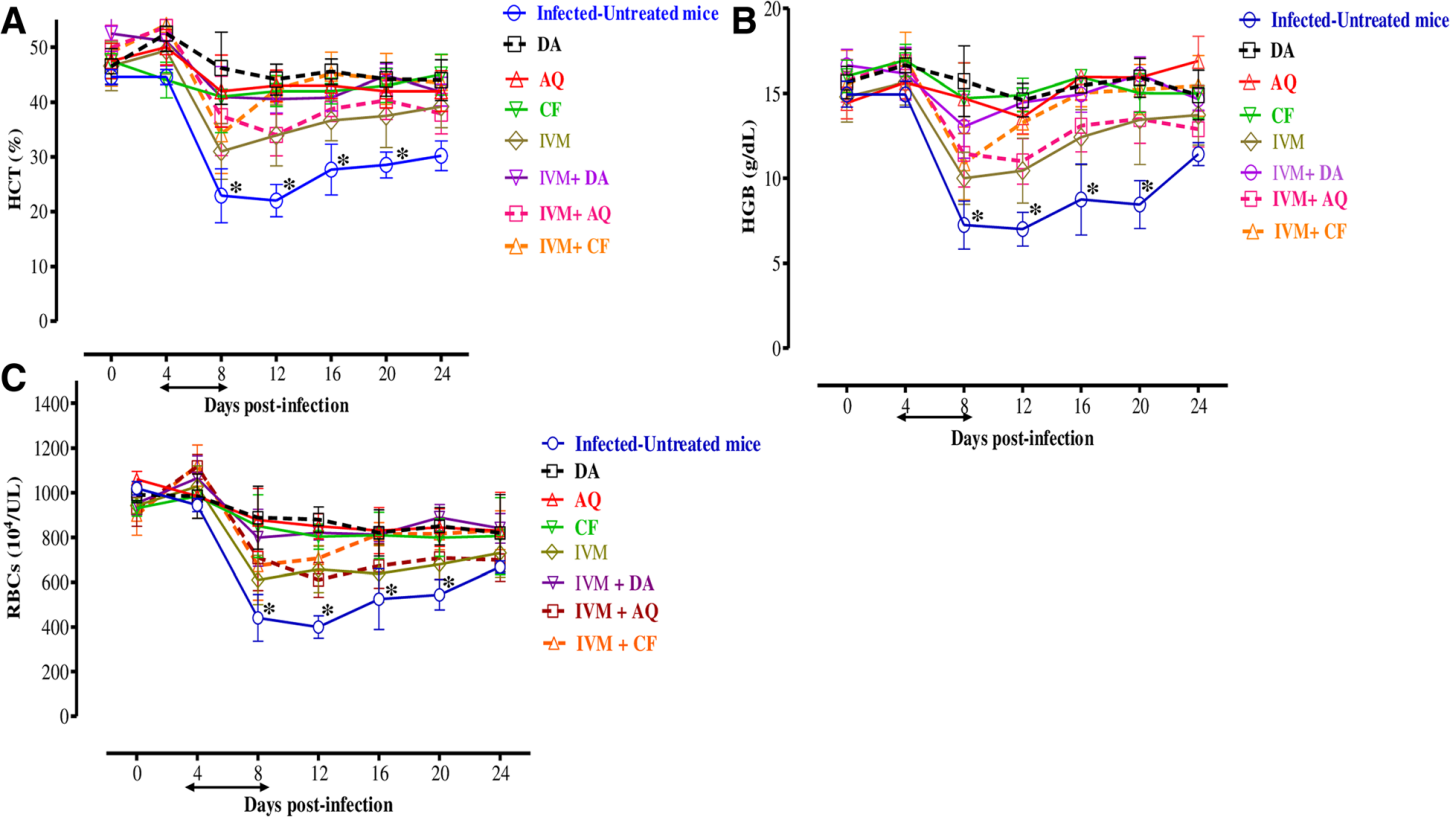
Changes in the hematocrit count (**a**), hemoglobin percentage (**b**), and red blood cell count (**c**) in IVM-treated mice in vivo. The graphs show changes in the hematocrit count (**a**), hemoglobin percentage (**b**), and red blood cell count (**c**) of mice treated as compared with untreated mice. The different groups used were DA-IP, AQ-oral, CF-oral, IVM-IP, IVM-DA, IVM-AQ, IVM-CF, and untreated mice. The values plotted are the mean ± standard deviation for two separate trials. Each group contained five mice. Asterisks (*) indicate statistical significance (*p* < 0.05) based on one-way ANOVA Tukey’s test using GraphPad Prism version 5.0 for Windows (GraphPad Software Inc., San Diego, CA, USA). The arrow indicates five consecutive days of treatment

**Fig. 6:**
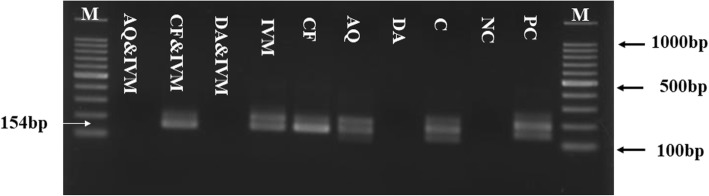
Molecular detection of parasite DNA in the treated groups**.** The image shows the molecular detection of parasites in the blood of treated groups on day 49. M is for the marker, NC is for the untreated-uninfected group that was used as a negative control, PC is for the untreated-infected group which used as the positive control, and C is for *B*. *microti* DNA control. The arrow shows the expected band length of 154 bp for positive cases of *B*. *microti*. The double bands observed with some of the positive controls represent amplicons of the first and second PCR. While a single band was observed in other groups due to the lower parasite DNA concentration

For further evaluation of IVM efficacy as compared with other drugs, the chemotherapeutic effect of IVM was examined in mice infected with *B*. *microti* (Fig. 3). In the DDW control group, the multiplication of *B*. *microti* increased significantly, reaching the highest parasitemia at 58.2% on day 8 post-infection (p.i). In all treated groups, the level of parasitemia was cleared at a significantly lower percentage of parasitemia than the control group (*p* < 0.05) from day’s 6 to 12 p.i. In mono-chemotherapy-treated mice, the peak parasitemia level reached 21.5%, 3.9%, and 4.3% on day 8 and 4.9% on day 7 in 4 mg/kg IVM, 25 mg/kg DA, 20 mg/kg AQ, and 20 mg/kg CF, respectively (Fig. 3). The parasitemia was undetectable in mice treated with 25 mg/kg DA, 20 mg/kg AQ, and 20 mg/kg CF via microscopy starting on day 13, 15, and 16 p.i., respectively. The parasitemia was undetectable by microscopy examination in mice treated with 4 mg/kg of IVM on day 30 p.i., while in the combination chemotherapy-treated groups, peak parasitemia levels reached 9.5%, 15%, and 14% in 2 mg/kg IVM–12.5 mg/kg DA on day 8, 2 mg/kg IVM–10 mg/kg CF on day 7, and 2 mg/kg IVM–10 mg/kg AQ on day 8, respectively (Fig. 4). Parasitemia was undetectable by microscopic examination in mice on days 13, 22, and 18 p.i. with 2 mg/kg IVM–12.5 mg/kg DA, 2 mg/kg IVM–10 mg/kg CF, and 2 mg/kg IVM–10 mg/kg AQ, respectively. Infection with *B*. *microti* reduced the hematocrit (HCT) count, hemoglobin (HGB) percentage, and red blood cell (RBC) count in mouse blood, as observed in the DDW control group on days 8, 12, 16, and 20 p.i. Significantly higher differences (*p* < 0.05) in HCT count, HGB percentage, and RBC count were observed between the DDW control group and all drug-treated groups on days 8, 12, 16, and 20 (Fig. 5a–c). Furthermore, parasite DNA was not detected in 25 mg/kg DA-IP, 2 mg/kg IVM–12.5 mg/kg DA, or 2 mg/kg IVM–10 mg/kg AQ on day 49. Meanwhile, in all other groups—20 mg/kg AQ–oral, 20 mg/kg CF–oral, 4 mg/kg IVM–IP, and 2 mg/kg IVM–10 mg/kg CF—parasite DNA was detected until day 49 (Fig. 6).

## Discussion

Although several reports documented the efficacy of IVM in the tick control programs to prevent the tick transmission of several *Babesia and Theileria species* to cattle and dogs [19, 20], this is the first study to evaluate the efficacy of IVM as an antiparasitic agent against *Babesia* and *Theileria* parasites in vitro and in vivo. In the current study, IVM inhibited the growth of *B*. *bovis*, *B*. *bigemina*, *B*. *divergens*, *B*. *caballi*, and *T*. *equi* in vitro. However, IVM showed slightly high IC_50_ values; they are still lower than those of *N*-acetyl l-cysteine [21], allicin [22], and clodinafop-propargyl against bovine *Babesia* and equine *Babesia/Theileria* parasites [23], and metronidazole and clindamycin phosphate against *B*. *gibsoni* [24]. Interestingly, several reports documented the effectiveness of IVM on *Plasmodium* which is one of the apicomplexan parasites closely related to *Babesia* and *Theileria* [25], and on *Leishmania*, and *Trypanosoma* parasites [15, 16]. This emphasizes that IVM is effective against many protozoan parasites.

Cytotoxicity studies showed that IVM was more likely to affect *Babesia* and *Theileria* than the host cells. This finding was consistent with that of Dou et al., who showed that IVM decreased the viability of breast cancer cell lines in a dose-dependent manner without cytotoxic activities on host cells [26].

Strikingly, the viability assay showed that IVM was more effective against *Babesia* than against *Theileria* parasites. *B*. *bovis*, *B*. *divergens*, *B*. *bigemina*, and *B*. *caballi* could not regrow with 4 × IC_50_ treatments of IVM, while *T*. *equi* could regrow at 4 × IC_50_ of IVM. This indicates that *T*. *equi* has better coping mechanisms to IVM treatment than *Babesia* parasites do. This finding is compatible with Reece et al., who reported that different malaria parasites species have used different coping mechanisms by changing in their investment in gametocytes during infections depending on their environment [27]. However, these patterns seem confusing; they can explain how parasites can respond to changes happened during infection. Therefore, *T*. *equi* might act by a different mechanism of action than that for *Babesia* species for coping the stress caused by IVM treatment. That may be one of the explanations of how can *T*. *equi* can be recovered again after the withdrawal of the drug pressure on the fourth day. However, we are unable to reach a definitive conclusion, since this study did not confirm the mode of action of IVM against *Babesia* and *Theileria* parasites. This finding is consistent with that of Tayebwa et al., who explained that *T*. *equi* might be affected through a different pathway than that for *Babesia* species [28].

Interestingly, combination chemotherapy has been recommended against drug-resistant protozoal and bacterial pathogens. Since, IVM is a well-known antiparasitic drug with a broad spectrum of activity, high efficacy, and it is used widely in the veterinary and human medicine [11, 12]. Additionally, the inhibition and cytotoxicity assays in the current study revealed that IVM showed higher IC_50_ values and lower selective index than currently available antibabesial drugs (DA, AQ, and CF). Therefore, the combination study aimed to enhance the potency of IVM while reducing the dose that led to reduced toxicity, subsequently reducing their toxic side effects. The in vitro combination treatment of IVM with DA, AQ, or CF revealed synergistic and additive effects against *Babesia* and *Theileria* parasites. Whereas, in the combination chemotherapy, drugs that share the same mode of action tend to yield a synergistic effect, which can be defined as the greater effect of two drugs in the combination than the sum of each drug when acting separately, or an additive effect, in which two drugs in the combination produce an effect equal to the sum of each drug when acting separately. Therefore, there is a need for further studies to reveal the exact mechanism of action of IVM against *Babesia* and *Theileria* and then, clarify the reason behind this synergetic and additive effect of the combined treatment with DA, AQ, and CF and choose the best combination to be used in the field.

The promising efficacy of IVM in vitro prompted us to evaluate its performance in vivo. IVM administered intraperitoneally resulted in an inhibitory effect in vivo but at lower rates than those with DA, AQ, and CF. IVM–DA, IVM–AQ, and IVM–CF combinations were evaluated in mice to determine whether combination treatment would reduce the dose needed of the single drugs without altering their therapeutic efficacy against *B*. *microti* infection. Interestingly, our results are compatible with Mendes et al., who revealed that oral administration of IVM at a concentration of 10 mg/kg resulted in 80% inhibition of *Plasmodium berghei* in vivo [29]. Additionally, Taman et al. documented that oral administration of IVM at a concentration of 25 mg/kg resulted in a significant reduction in *Schistosoma mansoni* female worms. While, administration of the same dose for two consecutive days revealed reduction of 45.4%, 27.6%, and 28.8% in female, male worms, and total worm burdens, respectively when compared to infected control groups receiving the vehicle only. Additionally, IVM showed higher chemotherapeutic effect than a high dose of praziquantel (six times higher dose than IVM), which is the reference drug used for the treatment of schistosomiasis [30]. Combination treatment of IVM–DA, IVM–AQ, and IVM–CF at half doses improved the efficacy of IVM at higher rates than those of monotherapy. The potentiation of IVM that was achieved in in vivo combination therapy confirmed the result that was observed in vitro, which draws attention to IVM as a good combinative drug. This finding is consistent with Canton et al., who reported ivermectin (IVM) and ricobendazole (RBZ) combination obtained significantly higher efficacy against IVM-resistant *Haemonchus* spp. than IVM and RBZ alone which have been proposed as a valid strategy to achieve effective nematode control in the presence of drug resistance [31].

In order to confirm the ability of IVM to eliminate *B*. *microti*, a PCR assay was performed on samples collected on day 49 p.i. Interestingly, this study confirmed the absence of *B*. *microti* DNA in groups treated with combination chemotherapy of IVM–DA and IVM–AQ. These results confirmed the importance of combination chemotherapy in the effective control of piroplasmosis. This finding further compels the need for combination therapy to achieve optimal efficacy and prevent relapse of infection or the development of a carrier state [32, 33]. Complimentarily, and consistent with a previous study (Udensi and Fagbenro-Beyioku), IVM did not show toxic side effects in mice [15]. Taken together, the findings advocate that IVM is a potential drug against human babesiosis as well as bovine and equine piroplasmosis. Even though IVM showed slightly low selective index, its combination with DA, AQ, and CF at lower concentrations than the single drugs could improve its effect and subsequently reduce its toxicity [32, 33].

## Conclusion

IVM showed efficacies on *Babesia* and *Theileria* consistent with efficacies reported on *P*. *falciparum*, *Leishmania*, and *Trypanosoma*. IVM effectiveness in vivo was comparable to that showed by DA and showed no toxic signs in mice. Therefore, IVM could be used as a chemotherapeutic drug for piroplasmosis. However, further studies are required to detect the exact mechanism of action of IVM on *Babesia* and *Theileria* parasites.

## Methods

### Cultivation conditions

#### Parasites and mice

The United States Department of Agriculture (USDA) strain of *T*. *equi* and *B*. *caballi*, the German bovine strain of *B*. *divergens* [32], the Texas strain of *B*. *bovis*, and the Argentina strain of *B*. *bigemina* were used for the in vitro studies [28]; for the in vivo studies, *B*. *microti* (Munich strain) was used [28, 34]. Eight-week-old female BALB/c mice (CLEA Japan, Inc., Tokyo, Japan) were housed in a pathogen-free environment with controlled temperature (22 °C), humidity, and a 12 h light/dark cycle, and used for cultivation of *B*. *microti* for in vivo studies.

#### Chemicals and reagents

Ivermectin (IVM), diminazene aceturate (DA), atovaquone (AQ), and clofazimine (CF) (Sigma-Aldrich, Japan) were prepared in dimethyl sulfoxide (DMSO) in stock solutions of 10 mM and stored at − 30 °C. A lysis buffer containing Tris-HCl (130 mM at pH 7.5), EDTA (10 mM), saponin (0.016% *w*/*v*), and Triton X–100 (1.6% *v*/*v*) was prepared, filtered through 0.22 μm of polyether sulfone, and stored at 4 °C to be mixed with 10,000× SYBR Green I (SGI) nucleic acid stain (Lonza, USA) 0.2 μL/mL before measuring the fluorescence.

#### In vitro cultivation of parasites

Purified equine or bovine red blood cells (RBCs) were used to maintain *B*. *caballi*, *T*. *equi*, *B*. *bovis*, *B*. *bigemina*, and *B*. *divergens*. A microaerophilic stationary-phase culture system at 37 °C, 5% CO_2_, 5% O_2_, and 90% N_2_ was used for the parasite cultivation as previously described [32]. For *B*. *bigemina* and *B*. *bovis* culture, medium 199 (M199; Sigma-Aldrich, Tokyo, Japan), supplemented with 40% bovine serum, was used for cultivation, while medium RPMI 1640 (Sigma-Aldrich, Tokyo, Japan) supplemented with 40% bovine serum was used to culture *B*. *divergens*. GIT (Sigma-Aldrich, Tokyo, Japan) supplemented with 40% equine serum was used to maintain *B*. *caballi* culture, while M199 supplemented with 40% equine serum and 13.6 μg/mL of hypoxanthine was used for *T*. *equi* cultivation. Further, 60 μg/mL of streptomycin and 0.15 μg/mL of amphotericin B were added to all of the culture media to prevent bacterial and fungal contamination. In vitro cultures of all five parasites were subcultured every 4 or 5 days to maintain good parasite growth.

### Cell cultures

Human foreskin fibroblast (HFF), Madin–Darby bovine kidney (MDBK) cell lines, and mouse embryonic fibroblasts (NIH/3 T3) were maintained in 75 cm^2^ culture flasks and incubated continuously at 37 °C in a humidified incubator with 5% CO_2_. Minimum Essential Medium Eagle (MEM; Gibco, Life Technologies, Grand Island, NY, USA) was used for MDBK cell cultivation, while Dulbecco’s modified Eagle’s medium (DMEM; Gibco, Life Technologies, Grand Island, NY, USA) was used for NIH/3 T3 and HFF cell cultivation. Each medium was supplemented with 10% fetal bovine serum, 50 μg/mL penicillin/streptomycin (Gibco, Life Technologies, Grand Island, NY, USA), and an additional 2 mM glutamine. The medium was changed every 3 to 4 days and incubated until approximately 80% confluent. The cells were stained with 4,6-diamidino-2-phenylindole dihydrochloride (DAPI; Sigma-Aldrich, St. Louis, MO, USA) to check mycoplasma-free contamination. After that, the cells were washed two times with Dulbecco’s phosphate-buffered saline (DPBS), and TrypLE Express (Gibco, Life Technologies, Grand Island, NY, USA) was used to allow cell detachment. Subsequently, viable cells were counted using a Neubauer improved C-Chip (NanoEn Tek Inc., Seoul, Korea) after staining with 0.4% Trypan blue solution.

### Cytotoxicity assay of IVM on HFF, MDBK, and NIH/3 T3 cell lines

The drug-exposure viability assay was performed in accordance with the protocol described previously [32]. Briefly, 100 μL of cells at a density of 5 × 10^4^ cells/mL was seeded per well and allowed to attach to a 96-well plate for 24 h at 37 °C in a humidified incubator with 5% CO_2_. For IVM, 10 μL of twofold dilutions was added to each well to a final concentration of 12.5 μM to 500 μM in triplicate, while for DA, AQ, and CF, 10 μL of twofold dilutions was added to each well, to a final concentration of 100 μM in triplicate. Wells with only a culture medium were used as blanks, while wells containing cells and a medium with 0.4% DMSO were used as a positive control. Subsequently, the plate was incubated for another 24 h. Ten microliters of Cell Counting Kit-8 (CCK-8) was added, and the plate was further incubated for 3 h; the absorbance was then measured at 450 nm using a microplate reader.

### The effects of IVM in host erythrocytes in vitro

The effects of IVM on bovine and equine RBCs were measured in accordance with the protocol previously described [28]. Briefly, bovine and equine RBCs were incubated in the presence of 10, 100, and 200 μM of IVM for 3 and 6 h at 37 °C. Afterward, the erythrocytes were washed thrice with drug-free media and used for the cultivation of *B*. *bovis*, *B*. *bigemina*, *B*. *divergens*, *B*. *caballi*, and *T*. *equi*. The untreated RBCs were used as a control. The effect was monitored using the fluorescence assay.

### Growth inhibitory effects in vitro

A fluorescent assay was used to determine the half-maximal inhibitory concentration (IC_50_) for IVM, DA, AQ, and CF as previously described [28, 34]. Briefly, IVM and CF in quantities of 1.56, 3.125, 6.25, 12.5, 25, 50, 100, 200, and 250 μM, as well as DA and AQ in quantities of 0.007, 0.015, 0.03, 0.06, 0.125, 0.25, 0.5, and 1 μM, were placed in a 96-well plate in triplicate to determine inhibition concentrations, with a 2.5% hematocrit for *B*. *bovis* and *B*. *bigemina* and a 5% hematocrit for *B*. *divergens*, *B*. *caballi*, and *T*. *equi*. Wells containing infected red blood cells (iRBCs) were used as positive controls, while wells with non-infected red blood cells (RBCs) were used as negative controls. The plate was incubated at 37 °C in a humidified multi-gas water-jacketed incubator with an atmosphere of 5% CO_2_, 5% O_2_, and 90% N_2_ for 96 h without changing media. After 96 h, 100 μL of lysis buffer containing SGI was directly added to each well and mixed gently by pipetting, wrapped in aluminum foil for protection from direct light, and incubated for 6 h at room temperature. The plate was then placed into the fluorescence spectrophotometer (Fluoroskan Ascent, Thermo LabSystems, Oceanside, California, USA). Relative fluorescence values were read at 485 and 518 nm for excitation and emission wavelengths, respectively. Gain values were set to percentages after subtraction of the mean values of the negative control and transferred into GraphPad Prism (GraphPad Software Inc. San Diego, CA, USA) to calculate the IC_50_ value using the nonlinear regression analysis (curve fit).

### Viability experiment in vitro

The assay was conducted in accordance with the protocol described previously [28]. Briefly, a 100 μL reaction volume, containing 90 μL of medium with different drug concentrations (0.25×, 0.5×, 1×, 2×, and 4× the IC_50_ of IVM and DA) and 10 μL of iRBCs adjusted to 1% parasitemia, was incubated in a 96-well microtiter plate at 37 °C in a humidified multi-gas water-jacketed incubator. The medium was changed daily for 4 days and replaced with new medium containing the same concentrations of drugs. In the course of treatment, Giemsa-stained thin blood smears were prepared, and the parasitemia was monitored every 12 h by counting the number of infected RBCs among 2000 RBCs. On day 5, 3 μL of treated RBCs from each well was mixed with 7 μL of fresh RBCs, transferred into a new 96-well microtiter plate, and cultured in drug-free medium. The medium was replaced every day, and the viability of drug-treated parasites was checked in Giemsa-stained thin blood smears 6 days after the last treatment. Parasitemia was calculated. The presence of parasites was recorded as positive (relapse), while no parasite was recorded as negative (total parasite clearance). Each experiment was performed in triplicate in three separate trials.

### Combination treatment of IVM with DA, AQ, and CF in vitro

The combination assay was conducted in accordance with the protocol previously described [32] in three separate trials. Three sets of duplicate wells with five selected concentrations, 0.25×, 0.5×, 1×, 2×, and 4× the IC_50_ of IVM with DA, AQ, and CF were cultivated in a 96-well plate (Additional file 1: Table S1). IVM single treatments were added to the first set of wells, while the second set of wells contained various concentrations of DA, AQ, or CF single treatments, and the third set contained the combinations of IVM with DA, AQ, or CF (IVM + DA, IVM + AQ, IVM + CF) at a constant ratio (1:1) [18]. One hundred microliter reaction volumes of media containing the drug concentrations and 2.5% and 5% hematocrits for *B*. *bovis* and *B*. *bigemina* and *B*. *divergens*, *B*. *caballi*, and *T*. *equi*, respectively, were cultivated for 4 days in a humidified incubator with 5% CO_2_, 5% O_2_, and 90% N_2_. On day 4, 100 μL of lysis buffer containing 2 × SG1 was added, and the plate was wrapped with aluminum foil for protection from light and incubated for 6 h at room temperature. Afterward, the plate was loaded into a fluorescence spectrophotometer, and the relative fluorescence values were read at 485 and 518 nm for excitation and emission wavelengths, respectively. The obtained fluorescence values were set to percentages after subtracting the mean values of the negative control. The growth inhibition values obtained were entered into Compusyn software for calculating the degree of association based on the combination index (CI) values. The CI values of the drug combination were determined using the formula [(1 × IC_50_) + (2 × IC_75_) + (3 × IC_90_) + (4 × IC_95_)]/10 (Additional file 2: Table S2), and the results were described as synergistic, additive, or antagonistic in accordance with the combination index scale: < 0.90, 0.90–1.10, and > 1.10, respectively [18].

### Chemotherapeutic effects of IVM against *B*. *microti*

The in vivo inhibitory effects of IVM were evaluated against *B*. *microti* in mice as previously described [28]. Briefly, *B*. *microti* recovered from frozen stock (stored at − 80 °C) was thawed and injected into two mice intraperitoneally. The parasitemia was monitored every day by microscopy, and when the parasitemia was over 30%, the mice were sacrificed, and blood was collected by cardiac puncture. After that, phosphate-buffered saline was used to dilute the blood to obtain an inoculum containing 1 × 10^7^/mL of *B*. *microti* iRBCs. Forty-five female 8-week-old BALB/c mice were caged in nine groups. The mice in group 1 were left uninfected to act as the negative control, while groups 2–9 were injected intraperitoneally (i.p.) with 0.5 mL of inoculum (1 × 10^7^ *B*. *microti* iRBCs). When the average parasitemia in all mice reached 1%, drug treatment was initiated for 5 days. The mice in group 2 were administered 0.2 mL of DDW intraperitoneally to act as the positive control, while groups 3 and 4 were i.p. injected with 0.2 mL of 25 mg/kg DA and 4 mg/kg of IVM single treatment, respectively. Groups 5 and 6 received 0.2 mL 20 mg/kg AQ and 20 mg/kg CF single treatment orally, respectively. Groups 7–9 were treated with combinations of IVM + DA, IVM + AQ, and IVM + CF, respectively. The parasitemia and hematocrit were monitored every 2 and 4 days for 45 days by microscopy and a hematology analyzer (Celltac α MEK-6450, Nihon Kohden Corporation, Tokyo, Japan), respectively. On day 49, all mice were anesthetized, and the blood was collected by cardiac puncture for PCR detection of parasites. The experiment was conducted two times. The significant differences between groups were determined by independent Student’s *t* test and one-way ANOVA Tukey’s test using GraphPad Prism version 5.0 for Windows (GraphPad Software Inc., San Diego, CA, USA). A *p* value of < 0.05 was considered statistically significant.

### Genomic DNA extraction and PCR detection of *B*. *microti*

A nested PCR (nPCR) targeting the *B*. *microti* small-subunit rRNA (ss-rRNA) gene was carried out in accordance with the previously described protocol [28, 35] after extracting the genomic DNA from the blood using a QIAamp DNA Blood Mini Kit (Qiagen, Tokyo, Japan). Briefly, a 10 μL reaction mixture containing 0.5 μM of each primer, 2 μL of 5 × SuperFi™ buffer, 0.2 mM dNTP mix, 0.1 μL of Platinum SuperFi™ DNA polymerase (Thermo Fisher Scientific, Tokyo, Japan), 1 μL of DNA template, and 4.9 μL of DDW was used to conduct the PCR amplification. The cycling conditions were 94 °C, 53 °C, and 72 °C for 30 s as denaturation, annealing, and extension steps for 35 cycles using the forward (5′-CTTAGTATAAGCTTTTATACAGC-3′) and reverse primer (5′-ATAGGTCAGAAACTTGAATGATACA-3′). Subsequently, under similar cycling conditions, 1 μL of DNA template from the first PCR amplification was used as the template for the nPCR assay using the forward (5′-GTTATAGTTTATTTGATGTTCGTTT-3′) and reverse primers (5′-AAGCCATGCGATTCGCTAAT-3′). The PCR products were then resolved by electrophoresis in a 1.5% agarose gel, stained with ethidium bromide, and visualized under the UV transilluminator. The bands with an expected size of 154 bp were considered positive.

### Statistical analysis

The IC_50_ values of IVM, DA, AQ, and CF were determined using the nonlinear regression curve fit in GraphPad Prism (GraphPad Software Inc., San Diego, CA, USA). Differences in parasitemia, hematology profiles, and body weight were analyzed using an independent Student’s *t* test and one-way ANOVA Tukey’s test using GraphPad Prism version 5.0 for Windows (GraphPad Software Inc., San Diego, CA, USA). A *p* value < 0.05 was considered statistically significant.

### Ethical clearance

All experiments were approved by the Animal Welfare Committee and performed in accordance with standards for the care and management of experimental animals as stipulated by Obihiro University of Agriculture and Veterinary Medicine (accession number of the animal experiment: 28–111-2/28–110).

## Additional files

Additional file 1: **Table S1.** Concentrations of ivermectin combined with diminazene aceturate and atovaquone against *Babesia* and *Theileria* parasites in vitro (DOCX 21 kb)

Additional file 2: **Table S2.** Calculation of weighted average of combination Index values (DOCX 17 kb)

Additional file 3: **Table S3.** The IC_50_ and selectivity index of DA, AQ, and CF (DOCX 19 kb)

## Abbreviations

AQ: Atovaquone
BW: Body weight
CF: Clofazimine
DA: Diminazene aceturate
EC_50_: Half-maximal effective concentration
HCT: Hematocrit
HFF: Human foreskin fibroblast
HGB: Hemoglobin
IC_50_: Half-maximal inhibitory concentration
IP: Intraperitoneally
IVM: Ivermectin
MDBK: Madin–Darby bovine kidney
NIH/3 T3: Mouse embryonic fibroblast
PCR: Polymerase chain reaction
RBCs: Red blood cells
RBZ: Ricobendazole
