# Supplementary material for: Evaluation of the inhibitory effect of ivermectin on the growth of Babesia and Theileria parasites in vitro and in vivo
Source: Trop Med Health. 2019 Jul 11;47:42. doi: 10.1186/s41182-019-0171-8 (PMC6625054; doi:10.1186/s41182-019-0171-8)
Supplement: Supplementary file 1 — Table S1. Concentrations of ivermectin combined with diminazene aceturate and atovaquone against Babesia and Theileria parasites in vitro (DOCX 21 kb) [file 41182_2019_171_MOESM1_ESM.docx]

**Table S1** Concentrations of ivermectin combined with diminazene aceturate and atovaquone against *Babesia* and *Theileria* parasites *in vitro*

| **Parasite** | **Concentration** | **Ivermectin** | **Diminazene aceturate** | **Atovaquone** | **Clofazimine** |
| --- | --- | --- | --- | --- | --- |
| ***B. bovis*** | **T_1_** | 13.325 | 0.0875 | 0.00975 | 2.06 |
|  | **T_2_** | 26.65 | 0.175 | 0.0195 | 4.12 |
|  | **T_3_** | 53.3 | 0.35 | 0.039 | 8.24 |
|  | **T_4_** | 106.6 | 0.7 | 0.078 | 16.48 |
|  | **T_5_** | 213.2 | 1.4 | 0.156 | 32.96 |
|  |  |  |  |  |  |
| ***B. bigemina*** | **T_1_** | 24.65 | 0.17 | 0.17525 | 1.4325 |
|  | **T_2_** | 49.3 | 0.34 | 0.3505 | 2.865 |
|  | **T_3_** | 98.6 | 0.68 | 0.701 | 5.73 |
|  | **T_4_** | 197.2 | 1.36 | 1.402 | 11.46 |
|  | **T_5_** | 394.4 | 2.72 | 2.804 | 22.92 |
|  |  |  |  |  |  |
| ***B. divergens*** | **T_1_** | 7.525 | 0.1075 | 0.0095 | 3.4625 |
|  | **T_2_** | 15.05 | 0.215 | 0.019 | 6.925 |
|  | **T_3_** | 30.1 | 0.43 | 0.038 | 13.85 |
|  | **T_4_** | 60.2 | 0.86 | 0.076 | 27.7 |
|  | **T_5_** | 120.4 | 1.72 | 0.152 | 55.4 |
|  |  |  |  |  |  |
| ***B. caballi*** | **T_1_** | 10.925 | 0.0055 | 0.0255 | 1.9875 |
|  | **T_2_** | 21.85 | 0.011 | 0.051 | 3.975 |
|  | **T_3_** | 43.7 | 0.022 | 0.102 | 7.95 |
|  | **T_4_** | 87.4 | 0.044 | 0.204 | 15.9 |
|  | **T_5_** | 174.8 | 0.088 | 0.408 | 31.8 |
|  |  |  |  |  |  |
| ***T. equi*** | **T_1_** | 22.525 | 0.775 | 0.02375 | 0.72 |
|  | **T_2_** | 45.05 | 0.355 | 0.0475 | 1.44 |
|  | **T_3_** | 90.1 | 0.71 | 0.095 | 2.88 |
|  | **T_4_** | 180.2 | 1.42 | 0.19 | 5.76 |
|  | **T_5_** | 360.4 | 2.84 | 0.38 | 11.52 |

Note: ^a^**T_1_–T_5_** refer to the concentrations (**µM**) of ivermectin combined with diminazene aceturate and atovaquone. Combined concentrations were based on the calculated IC_50_ values obtained from the *in vitro* fluorescence-based assay
