# Supplementary material for: Evaluation of the inhibitory effect of ivermectin on the growth of Babesia and Theileria parasites in vitro and in vivo
Source: Trop Med Health. 2019 Jul 11;47:42. doi: 10.1186/s41182-019-0171-8 (PMC6625054; doi:10.1186/s41182-019-0171-8)
Supplement: Supplementary file 2 — Table S2. Calculation of weighted average of combination Index values (DOCX 17 kb) [file 41182_2019_171_MOESM2_ESM.docx]

| **Weighted average CI values ^b^** | **Combination index values at** | | | | **Drug combinations ^a^** | **Parasites** |
| --- | --- | --- | --- | --- | --- | --- |
|  | **IC_95_** | **IC_90_** | **IC_75_** | **IC_50_** |  |  |
| **1.11051**  **1.04737** | 1.007  1.0022 | 1.0432  1.0327 | 1.0623  0.9252 | 1.8229  0.7164 | **IVM + DA**  **IVM + AQ** | ***B. bovis*** |
| **0.92317** | 0.9036 | 0.8686 | 0.9894 | 1.0327 | **IVM + CF** |  |
| **0.40539**  **0.82283** | 0.1997  0.8714 | 0.2995  0.7052 | 0.5903  0.8477 | 1.1760  0.9317 | **IVM + DA**  **IVM + AQ** | ***B. bigemina*** |
| **1.01390** | 0.9228 | 1.0272 | 0.9714 | 1.4234 | **IVM + CF** |  |
| **0.01779**  **1.04901** | 0.0613  0.9337 | 0.0682  1.0193 | 0.0059  1.0728 | 0.2837  1.5526 | **IVM + DA**  **IVM + AQ** | ***B. divergens*** |
| **1.01372** | 0.9876 | 0.9897 | 1.2869 | 0.6439 | **IVM + CF** |  |
| **0.14328**  **1.07398** | 0.1385  1.0172 | 0.1605  1.3712 | 0.1018  1.0512 | 0.1937  0.4550 | **IVM + DA**  **IVM + AQ** | ***B. caballi*** |
| **1.10847** | 1.1109 | 1.1191 | 1.1812 | 0.9214 | **IVM + CF** |  |
| **1.10124**  **1.05367** | 1.1227  1.0710 | 0.9011  1.0501 | 1.2732  1.0207 | 1.2719  1.0610 | **IVM + DA**  **IVM + AQ** | ***T. equi*** |
| **0.99644** | 0.8803 | 0.9767 | 1.1692 | 1.1747 | **IVM + CF** |  |

**Table S2** Calculation of weighted average of combination Index values

^a^ Two-drug combination between IVM with DA, AQ and CF at a concentration of approximately 0.25 x IC_50_, 0.5 x IC_50_, IC_50_, 2 x IC_50_, and 4 x IC_50_ (constant ratio). ^b^ The weighted average CI value was calculated with the formula [(1 x IC_50_) + (2 x IC_75_) + (3 x IC_90_) + (4 x IC_95_)]/10

*Abbreviations:* *CI* combination index value, *IC_50_* 50% inhibition concentration, *IVM* ivermectin, *DA* diminazene aceturate, *AQ* atovaquone, *CF* clofazimine
