# Supplementary material for: Evaluation of the inhibitory effect of ivermectin on the growth of Babesia and Theileria parasites in vitro and in vivo
Source: Trop Med Health. 2019 Jul 11;47:42. doi: 10.1186/s41182-019-0171-8 (PMC6625054; doi:10.1186/s41182-019-0171-8)
Supplement: Supplementary file 3 — Table S3. The IC50 and selectivity index of DA, AQ, and CF (DOCX 19 kb) [file 41182_2019_171_MOESM3_ESM.docx]

**Table S3** The IC_50_ and selectivity index of DA, AQ, and CF

| **Compound** | ***Babesia* and *Theileria*** | **IC_50_ (µM)^a^** | **EC_50_ (µM)^b^** | | | **Selective index^c^** | | |
| --- | --- | --- | --- | --- | --- | --- | --- | --- |
|  |  |  | **MDBK** | **NIH/3T3** | **HFF** | **MDBK** | **NIH/3T3** | **HFF** |
| **DA** | *B. bovis* | **0.35 ± 0.06** | **˃100** | **˃100** | **˃100** | **˃ 285.7** | **˃ 285.7** | **˃ 285.7** |
|  | *B. bigemina* | **0.68 ± 0.09** | **˃100** | **˃100** | **˃100** | **˃ 147.1** | **˃ 147.1** | **˃ 147.1** |
|  | *B. divergens* | **0.43 ± 0.05** | **˃100** | **˃100** | **˃100** | **˃ 232.5** | **˃ 232.5** | **˃ 232.5** |
|  | *B. caballi* | **0.02 ± 0.0002** | **˃100** | **˃100** | **˃100** | **˃ 5000** | **˃ 5000** | **˃ 5000** |
|  | *T. equi* | **0.71 ± 0.05** | **˃100** | **˃100** | **˃100** | **˃ 140.8** | **˃ 140.8** | **˃ 140.8** |
| **AQ** | *B. bovis* | **0.039 ± 0.002** | **˃100** | **˃100** | **˃100** | **˃ 2564.1** | **˃ 2564.1** | **˃ 2564.1** |
|  | *B. bigemina* | **0.701 ± 0.04** | **˃100** | **˃100** | **˃100** | **˃ 142.7** | **˃ 142.7** | **˃ 142.7** |
|  | *B. divergens* | **0.038 ± 0.002** | **˃100** | **˃100** | **˃100** | **˃ 2631.6** | **˃ 2631.6** | **˃ 2631.6** |
|  | *B. caballi* | **0.102 ± 0.014** | **˃100** | **˃100** | **˃100** | **˃ 980.4** | **˃ 980.4** | **˃ 980.4** |
|  | *T. equi* | **0.095 ± 0.065** | **˃100** | **˃100** | **˃100** | **˃ 1052.6** | **˃ 1052.6** | **˃ 1052.6** |
| **CF** | *B. bovis* | **8.24 ± 1.7** | **34.7± 3.4** | **˃100** | **˃100** | **4.2** | **˃ 12.1** | **˃ 12.1** |
|  | *B. bigemina* | **5.73 ± 1.9** | **34.7± 3.4** | **˃100** | **˃100** | **6.1** | **˃ 17.5** | **˃ 17.5** |
|  | *B. divergens* | **13.85 ± 4.3** | **34.7± 3.4** | **˃100** | **˃100** | **2.5** | **˃ 7.2** | **˃ 7.2** |
|  | *B. caballi* | **7.95 ± 1.8** | **34.7± 3.4** | **˃100** | **˃100** | **4.4** | **˃ 12.6** | **˃ 12.6** |
|  | *T. equi* | **2.88 ± 0.9** | **34.7± 3.4** | **˃100** | **˃100** | **12.1** | **˃ 34.7** | **˃ 34.7** |

^a^ Half-maximal inhibition concentration of diminazene aceturate (DA), atovaquone (AQ), and clofazimine (CF) on the *in vitro* culture of parasites. The value was determined from the dose-response curve using nonlinear regression (curve fit analysis). The values are the means of experiments run in triplicate.

^b^ Half-maximal effective concentration of AQ, DA, and CF on cell lines. The values were determined from the dose-response curve using nonlinear regression (curve fit analysis). The values are the means of experiments in triplicate.

^c^ Ratio of the EC_50_ of cell lines to the IC_50_ of each species. High numbers are favorable.

*Abbreviations: DA* diminazene aceturate, *AQ* atovaquone, *CF* clofazimine, *MDBK* Madin–Darby bovine kidney, *NIH/3T3* Mouse embryonic fibroblast, *HFF* Human foreskin fibroblast
